# Temporal Dynamics of the Ruminant Type I IFN-Induced Antiviral State against Homologous Parainfluenza Virus 3 Challenge In Vitro

**DOI:** 10.3390/v14051025

**Published:** 2022-05-11

**Authors:** Min Sun, Fei Hao, Wenliang Li, Zilong Cheng, Wenwen Zhang, Leilei Yang, Li Mao, Maojun Liu

**Affiliations:** 1 Institute of Veterinary Medicine, Jiangsu Academy of Agricultural Sciences, Nanjing 210014, China; 20140010@jaas.ac.cn (F.H.); 20210005@jaas.ac.cn (Z.C.); 20110978@jaas.ac.cn (W.Z.); 20120034@jaas.ac.cn (L.Y.); 20100014@jaas.ac.cn (L.M.); 20030022@jaas.ac.cn (M.L.); 2 School of Life Sciences, Jiangsu University, Zhenjiang 212013, China; 3 School of Food and Biological Engineering, Jiangsu University, Zhenjiang 212013, China

**Keywords:** goat interferon-α, dynamic reaction, caprine parainfluenza virus 3, antiviral activity, goat interferon-τ, bovine parainfluenza virus 3

## Abstract

Viruses have evolved diverse strategies to evade the antiviral response of interferons (IFNs). Exogenous IFNs were applied to eliminate the counteracting effect and possess antiviral properties. Caprine parainfluenza virus 3 (CPIV3) and bovine parainfluenza virus type 3 (BPIV3) are important pathogens associated with respiratory diseases in goat and cattle, respectively. To explore the feasibility of type I IFNs for control of CPIV3 and BPIV3 infection, the activated effects of IFN-stimulated genes (ISGs) and the immunomodulation responses of goat IFN-α were detected by transcriptomic analysis. Then, the antiviral efficacy of goat IFN-α and IFN-τ against CPIV3 and BPIV3 infection in MDBK cells was evaluated using different treatment routes at different infection times. The results showed that CPIV3 infection inhibited the production of type I IFNs, whereas exogenous goat IFN-α induced various ISGs, the IFN-τ encoding gene, and a negligible inflammatory response. Consequently, goat IFN-α prophylaxis but not treatment was found to effectively modulate CPIV3 and BPIV3 infection; the protective effect lasted for 1 week, and the antiviral activity was maintained at a concentration of 0.1 μg/mL. Furthermore, the antiviral activity of goat IFN-τ in response to CPIV3 and BPIV3 infection is comparable to that of goat IFN-α. These results corroborate that goat IFN-α and IFN-τ exhibit prophylactic activities in response to ruminant respiratory viral infection in vitro, and should be further investigated for a potential use in vivo.

## 1. Introduction

Interferons (IFNs) are cytokines that act upon cells to impart resistance to virus replication, and the IFN family contains type I, II, and III IFNs [1,2]. IFN-α belongs to the type I IFN subfamily, and can be divided into several subtypes, which display individual antiviral properties [3,4]. Currently, the IFN-α proteins encoded by humans and other animal species are all highly effective in limiting virus replication and spread [4,5,6,7]. Although IFNs are broad-spectrum antiviral agents, the sequence conservation of the IFN-α proteins from different species can differ considerably [8,9], and there are also differences in the potency of different IFN-a homologues [10,11]. The antiviral property of goat IFN-α has not been evaluated for prophylaxis and/or therapeutic treatment of viral infections to date.

Upon type I IFNs binding to cell surface receptors IFNAR1 and IFNAR2, the JAK/STAT pathway is activated to induce the expression of IFN-stimulated genes (ISGs), which possess diverse antiviral properties [12]. Meanwhile, the IFNs stimulate the intrinsic host antiviral response, which provides an efficient and reduced-risk antiviral strategy. Furthermore, with several viruses having developed efficient strategies to antagonize the production of IFNs [13,14], exogenous application of purified IFNs could eliminate these counteracting effects and possess antiviral properties. For example, the PEGylated IFN-α2 has been successful in clinical applications for the treatment of chronic virus infections [15].

Caprine parainfluenza virus 3 (CPIV3) and bovine parainfluenza virus type 3 (BPIV3) both belong to the respirovirus genus of the *Paramyxoviridae* family, and are associated with respiratory diseases in goats and cattle, respectively [16]. CPIV3 can effectively infect primary goat tracheal epithelial cells and induce pathological lesions mainly on the lungs and trachea [17,18]. BPIV3 is an important pathogen associated with bovine respiratory disease complex (BRDC), causing serious economic losses worldwide [19]. To date, no specific treatment for CPIV3 and BPIV3 infection is available, and both viruses are reported to escape from host antiviral responses [18,20,21], whereas the potential of exogenous goat IFN-α against CPIV3 and BPIV3 infection has not been investigated to date.

The aim of this study is to determine the antiviral activities of goat IFN-α against CPIV3 and BPIV3 by investigating the dynamics of exogenous goat IFN-α action in MDBK cells. The results show that goat IFN-α could lead to dramatic changes in cellular properties with the upregulation of 550 genes, confirming the utility of goat IFN-α as a prophylactic to modulate CPIV3 and BPIV3 infection. This study provides novel insights for future clinical applications using goat IFN-α.

## 2. Materials and Methods

### 2.1. Cells and Viruses

Madin–Darby bovine kidney (MDBK) cells were purchased from the China Institute of Veterinary Drug Control and grown in Dulbecco’s modified eagle medium (DMEM, Hyclone, Shanghai, China) supplemented with 10% heat-inactivated fetal bovine serum (FBS). CPIV3 strain JS2013 and BPIV3 strain TL2021 were isolated and stored in our laboratory.

### 2.2. Protein Prokaryotic Expression and Purification

Goat IFN-α (NM_001285704.1, Detai Biologics, Nanjing, China) and goat IFN-τ (KJ764650.1, Zoonbio Biotechnology, Nanjing, China) proteins were purified by Ni-sepharose and affinity purification. The purified proteins were identified with SDS-PAGE and Western blot assay. The goat IFN-α and IFN-τ proteins were stored at −70 °C at 0.2 mg/mL and 0.25 mg/mL, respectively.

### 2.3. SDS-PAGE and Western Blot Assay

The total cellular proteins were extracted with RIPA lysis and extraction buffer (Thermo Fisher, Shanghai, China) with protease inhibitor PMSF. Each sample was subjected to SDS-PAGE, and prepared for Western blot as described previously [18]. Anti-β-actin (proteintech, Wuhan, China) or CPIV3-specific mAbs 5E4 were used as the primary antibodies. HRP-conjugated goat anti-mouse (IgG) was used as secondary antibody. The results were detected by enhanced chemiluminescence reagents (Vazyme, Nanjing, China) and analyzed with an automatic chemiluminescence imaging analysis system (TANON, Shanghai, China).

### 2.4. RT-qPCR

Total cellular RNA was extracted using RNAiso Plus reagent (Takara, Dalian, China) according to the manufacturer’s protocols. cDNA synthesis was performed with a PrimeScript RT reagent kit (Takara, Dalian, China). The expression level of the specific genes was determined with SYBR^®^ Premix Ex Taq™ II (Takara, Dalian, China), and the β-Actin gene was used as the internal control. The relative quantities of mRNA accumulation were calculated based on the 2^−ΔΔCt^ method. CPIV3 RNA copies in the cellular supernatant were measured as described previously [22]. The gene-specific primers for qPCR are listed in Table S1. qPCR amplification was carried out on an Applied Biosystems Step One^TM^ real-time PCR system.

### 2.5. Virus Titration

MDBK cells were seeded in a 96-well cell plate and cultured overnight. The virus supernatant was 10-fold serially diluted, then added to the MDBK cells, and the uninfected cells were used as the negative control. Cellular changes were observed daily for one week, and the cells showing a cytopathic effect (CPE) were noted as positive for viral growth. The 50% tissue culture infective dose (TCID_50_) was calculated by the Reed–Muench method [23].

### 2.6. Transcriptomic Analysis

Total cellular RNA was extracted from the goat IFN-α-incubated or untreated MDBK cells with RNAiso Plus reagent (Takara, Dalian, China) according to the manufacturer’s instructions. RNA quality was assessed and sequenced using Illumina Novaseq6000 by Gene Denovo Biotechnology Co. (Guangzhou, China). Then, high-quality clean reads were mapped to the reference genome (ncbi_GCF_002263795.2), and the mRNA differential expression analysis was performed with DESeq2 software. The genes/transcripts with the parameter of false discovery rate (FDR) below 0.05 and absolute fold change ≥ 2 were considered differentially expressed genes/transcripts. Two replicates were set for each sample.

### 2.7. Statistical Analysis

The statistical analysis was constructed with GraphPad Prism 6, and *p* values less than 0.05 were considered statistically significant. Statistical significance is reflected with an asterisk; (*), *, *p* < 0.05, **, *p* < 0.01.

## 3. Results

### 3.1. CPIV3 Infection Inhibited the Activation of Endogenous Type I IFNs

**Figure 1:**
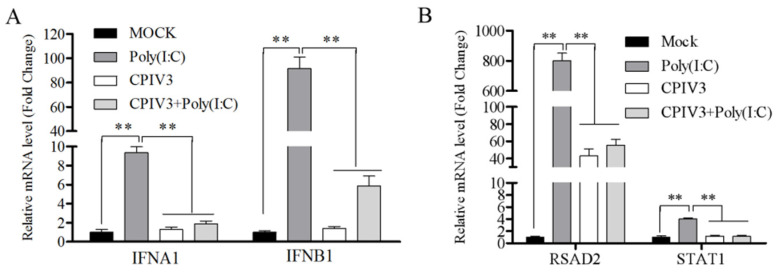
CPIV3 infection inhibited the activation of endogenous type I IFNs in MDBK cells. (**A**) CPIV3 infection inhibited the transcription of *IFNA1* and *IFNB1* in virus-infected and poly(I:C)-stimulated cells. (**B**) CPIV3 infection inhibited mRNA levels of *RSAD2* and *STAT1* stimulated by poly(I:C). MDBK cells were infected with CPIV3 at an MOI of 1 for 48 h, poly(I:C) transfection was used as a positive control, and the uninfected cells were used as the negative control. Then, CPIV3-infected cells or CPIV3-infected poly(I:C)-transfected cells were harvested, and the relative quantitation values for *IFNA1*, *IFNB1*, *RSAD2*, and *STAT1* mRNA were determined by qPCR assay. Error bars indicate standard error of mean (SEM) of two independent experiments. **, *p* < 0.01.

CPIV3 infection antagonized the JAK/STAT pathway, and nonstructural protein C was an important antagonistic protein [18]. Here, the RT-qPCR results show that CPIV3 infection failed to induce the activation of type I IFN genes (*IFNA1* and *IFNB1*), which was in accordance with the previous transcriptomic analysis (Figure 1A). Furthermore, CPIV3 infection significantly inhibited the transcription of the ISGs (*RSAD2* and *STAT1*) in poly(I:C)-stimulated cells (Figure 1B), which both implied that CPIV3 infections have evolved efficient strategies to antagonize the induction of endogenous type I IFNs.

### 3.2. Exogenous Goat IFN-α Stimulated the Production of Various ISGs

As CPIV3 is widely prevalent in goat and sheep in China [24,25], here, we characterized the immune response of goat IFN-α. CPIV3 proliferated effectively and yielded a high viral titer in MDBK cells; thus, MDBK cells could be an ideal cell model to assess CPIV3 infection levels. Due to the silence of endogenous IFN production in CPIV3-infected cells, we were next interested in determining the changes in cellular properties of exogenous goat IFN-α in MDBK cells. Transcriptomic analysis showed that goat IFN-α induced the upregulation of 550 genes in MDBK cells (fold change ≧ 2) (Table S2). Here, the top ten most upregulated genes were *SERPINB2*, *IFI27*, *RSAD2*, *OAS2*, *OAS1Z, PLAC8*, *GBP2*, *IFI44*, *IFI44L*, and *GNGT2*, which had mostly been shown to be antiviral [26,27,28,29,30]. Meanwhile, ISGs had been reported to take on a wide range of activities [2]. Here, goat IFN-α induced the upregulation of the negative regulatory factors *SOCS1* and *USP18,* and the positive regulatory factors *IFI6*, *cGAS*, *TLR3*, and *STAT1*. The direct antiviral ISGs acted on different stages of virus replication [1]; the ISGs that had been reported to affect virus entry into cells (*Mx1*, *Mx2*, *CH25H*, and *IFITM1/3*), inhibit virus translation and replication (*ISG15*, *IFIT1/3/5*, and *OAS1/2*), and inhibit viral egress (*RSAD2* and *BST2*) were all upregulated in goat IFN-α-induced MDBK cells. Furthermore, we employed RT-qPCR to validate the expression profile of partial genes in goat IFN-α-incubated MDBK cells, and the results were basically the consistent with the transcriptome results (Figure S1).

### 3.3. Goat IFN-α Prophylaxis but Not Treatment Modulated CPIV3 Infection

**Figure 2:**
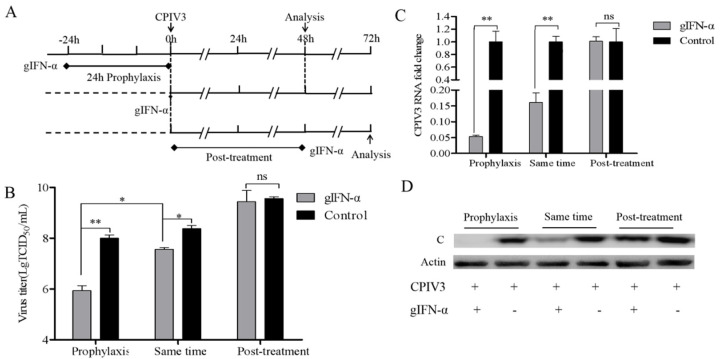
Goat IFN-α prophylaxis but not treatment modulates CPIV3 infection in MDBK cells. (**A**) Experimental schematic. MDBK cells were treated with goat IFN-α (1 μg/mL) 24 h before infection or together with infection, and then the samples were harvested at 48 hpi. MDBK cells were treated with goat IFN-α (1 μg/mL) 48 h after infection, and the samples were harvested 24 h later. The MDBK cells were infected with CPIV3 strain JS2013 at an MOI of 1. (**B**) TCID_50_ results, detecting the virus titer in the culture supernatant between different groups. (**C**) The viral RNA level enabled quantification of the viral shedding in the culture supernatant by RT-qPCR. (**D**) The expression level of CPIV3 nonstructural protein C was detected by Western blot with the anti-CPIV3 protein C antibody. The untreated cells were used as the negative control. The results were reported based on duplicate independent experiments and compared with the negative control in each group. *, *p* < 0.05, **, *p* < 0.01.

To assess the precise impact of IFN-α on CPIV3 infection, we added 1 μg/mL of exogenous goat IFN-α together with the CPIV3 infection and incubated the mixture for a further 48 h (Figure 2A). This treatment reduced the viral titer by about 6.5-fold (Figure 2B), and the viral RNA release into the supernatant was also affected (Figure 2C). To model prophylaxis, 1 μg/mL of exogenous goat IFN-α was added to MDBK cells 24 h before infection (Figure 2A). The viral titer was significantly reduced by about 115-fold (Figure 2B), and the shedding of viral RNA into the culture supernatant was reduced by 95% (Figure 2C). As CPIV3 nonstructural protein C was detected at 48 hpi and the mAb 5E4 was highly specific, in our studies of the antiviral activity of CPIV3 infection in response to goat IFN-α, we established a CPIV3 nonstructural protein C-based immunoblotting assay referring to that of HPIV3 infection [31]. The results showed that the expression of CPIV3 nonstructural protein C was significantly downregulated, as the goat IFN-α incubated before or with the CPIV3 infection on MDBK cells (Figure 2D). In contrast, treatment of exogenous goat IFN-α after CPIV3 infection was not effective in modulating CPIV3 infection, either as characterized by the viral titer, viral RNA load, or expression level of nonstructural protein C (Figure 2).

### 3.4. CPIV3 Infection Was Sensitive to Exogenous Goat IFN-α

**Figure 3:**
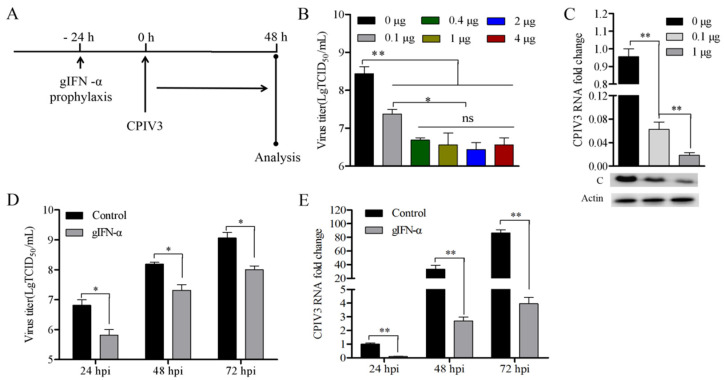
Sensitivity of CPIV3 infection to the antiviral activity of goat IFN-α in MDBK cells. (**A**) Experimental schematic. MDBK cells were treated with different concentrations goat IFN-α (0.1, 0.4, 1, 2, or 4 μg/mL) 24 h before infection, and the untreated cells were used as the negative control. Then, MDBK cells were infected with CPIV3 strain JS2013 at an MOI of 1, and the samples were harvested at 48 hpi. (**B**) TCID_50_ results, detecting the virus titer in the culture supernatant between different groups. (**C**) Viral RNA level and the expression level of CPIV3 protein C in goat IFN-α (0.1 or 1 μg/mL)-incubated or untreated cells were quantified by RT-qPCR and Western blot assay, respectively. (**D**) TCID_50_ results and viral RNA level (**E**), detecting the virus titer and RNA copies in the culture supernatant at the indicated time (24 hpi, 48 hpi, and 72 hpi). The cells were incubated with 0.1 μg/mL goat IFN-α prior to CPIV3 infection and the viral titer in untreated cells of each group, and the RNA copies in untreated cells at 24 hpi were used as the negative control. *, *p* < 0.05, **, *p* < 0.01.

To further explore the antiviral activity of goat IFN-α in response to CPIV3 infection, different concentrations of goat IFN-α were preincubated in MDBK cells for 24 h, and then antiviral assays were performed to determine the sensitivity (Figure 3A). Higher concentrations of exogenous goat IFN-α (up to 4 μg/mL) significantly modulated CPIV3 infection and showed no significant difference compared to a concentration of 0.4 μg/mL, which indicates the ultimate antiviral activity of goat IFN-α in response to CPIV3 (Figure 3B). With a concentration of 0.1 μg/mL of goat IFN-α, the viral titer was reduced by about 11.5-fold at 48 hpi (Figure 3B). CPIV3 RNA copies and the expression level of CPIV3 protein C were also reduced significantly, although the antiviral activity of 0.1 μg/mL goat IFN-α was lower than that of 1 μg/mL (Figure 3C). Furthermore, goat IFN-α (0.1 μg/mL) inhibited CPIV3 replication (Figure 3D) and reduced RNA copies (Figure 3E) in the whole infection process.

### 3.5. One Week after Administration, Exogenous Goat IFN-α Still Modulated CPIV3 Infection

**Figure 4:**
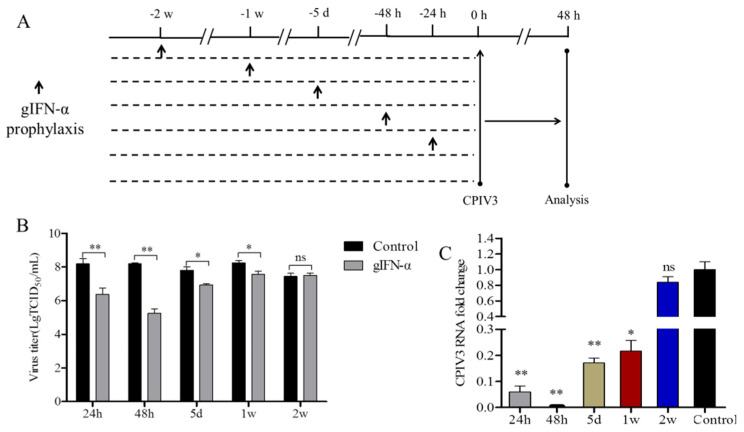
Duration of the antiviral activity of goat IFN-α in response to CPIV3 infection. (**A**) Experimental schematic. MDBK cells were treated with goat IFN-α (1 μg/mL), and cells were subsequently cultured for varying lengths of time (0 h, 24 h, 48 h, 5 d, 1 w, or 2 w) prior to infection. The untreated cells were used as the negative control. MDBK cells were then infected with CPIV3 strain JS2013 at an MOI of 1, and the samples were harvested at 48 hpi. (**B**) TCID_50_ results, detecting the virus titer in the culture supernatant between different groups. (**C**) The viral RNA level enabled quantification of viral shedding in the culture supernatant by RT-qPCR. *, *p* < 0.05, **, *p* < 0.01.

To further investigate the duration of the goat IFN-α response, MDBK cells were incubated with goat IFN-α (1 μg/mL) and cultured for up to a further 2 weeks before infection (Figure 4A). When goat IFN-α was administered 24 h before infection, the viral titer was reduced by about 65-fold. The antivirus effect was still promoted with goat IFN-α incubation for 48 h, as the viral titer was reduced about 274-fold. Then, the level of inhibition was reduced but still statistically significant 5 days and 1 week after goat IFN-α incubation, as the viral titer was reduced about by 7.5-fold and 5-fold, respectively. However, this effect was lost 2 weeks after goat IFN-α incubation (Figure 4B). Meanwhile, these dynamics were similar for the CPIV3 RNA copies. The shedding of viral RNA into the culture supernatant was reduced by 94%, 99%, 83%, 78%, and 16% with the goat IFN-α incubated prior to CPIV3 infection for 24 h, 48 h, 5 d, 1 w, and 2 w, respectively (Figure 4C). 

### 3.6. Goat IFN-τ Inhibited CPIV3 Infection

**Figure 5:**
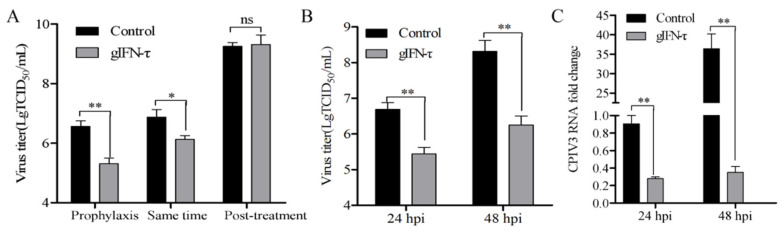
Goat IFN-τ inhibits CPIV3 infection in MDBK cells. (**A**) Goat IFN-τ prophylaxis but not treatment modulates CPIV3 infection in MDBK cells. MDBK cells were treated with goat IFN-τ (1 μg/mL) 24 h before infection or together with infection, and then the samples were harvested at 24 hpi. MDBK cells were treated with goat IFN-τ (1 μg/mL) 48 h after infection, and the samples were harvested 24 h later. The MDBK cells were infected with CPIV3 strain JS2013 at an MOI of 1. Then, viral titer was detected with TCID_50_ assay. (**B**) TCID_50_ results. MDBK cells were treated with goat IFN-τ (1 μg/mL) 24 h before infection, and the untreated cells were used as the negative control. Then, the culture supernatant was collected at 24 hpi and 48 hpi. (**C**) The viral RNA level enabled quantification of viral shedding in the culture supernatant by RT-qPCR. MDBK cells were treated with goat IFN-τ (1 μg/mL) 24 h before infection; then, the culture supernatant was collected at 24 hpi and 48 hpi. The viral RNA level in untreated cells at 24 hpi was used as the control. *, *p* < 0.05, **, *p* < 0.01.

IFN-τ, encoded by the *IFNT1* gene, is produced only by ruminants but exhibits broad cross-species antiviral activity. Here, exogenous goat IFN-α did not induce endogenous *IFNA1*, *IFNB1*, or *IFNL3* mRNA expression, but *IFNT1* was significantly increased by about 2.5-fold (Table S2). We then we tested the antiviral activity of goat IFN-τ in response to CPIV3. MDBK cells were infected with CPIV3 either prior to, together with, or after goat IFN-τ (1 μg/mL) stimulation. The TCID_50_ results showed that the incubation of goat IFN-τ prior to or together with CPIV3 infection significantly inhibited the replication of CPIV3, with the viral titer reduced by about 18-fold and 6-fold at 24 hpi, respectively, whereas there was no antiviral activity on an already-established infection (Figure 5A). To further investigate the antiviral activity of goat IFN-τ in response to CPIV3, MDBK cells were incubated with goat IFN-τ (1 μg/mL) for 24 h; then, the culture supernatant was collected at 24 hpi and 48 hpi. The viral titer was reduced by about 18-fold and 115-fold, respectively (Figure 5B). CPIV3 RNA copies were also reduced significantly following the incubation of goat IFN-τ (Figure 5C).

### 3.7. Exogenous Goat IFN-α and IFN-τ Inhibited BPIV3 Replication

**Figure 6:**
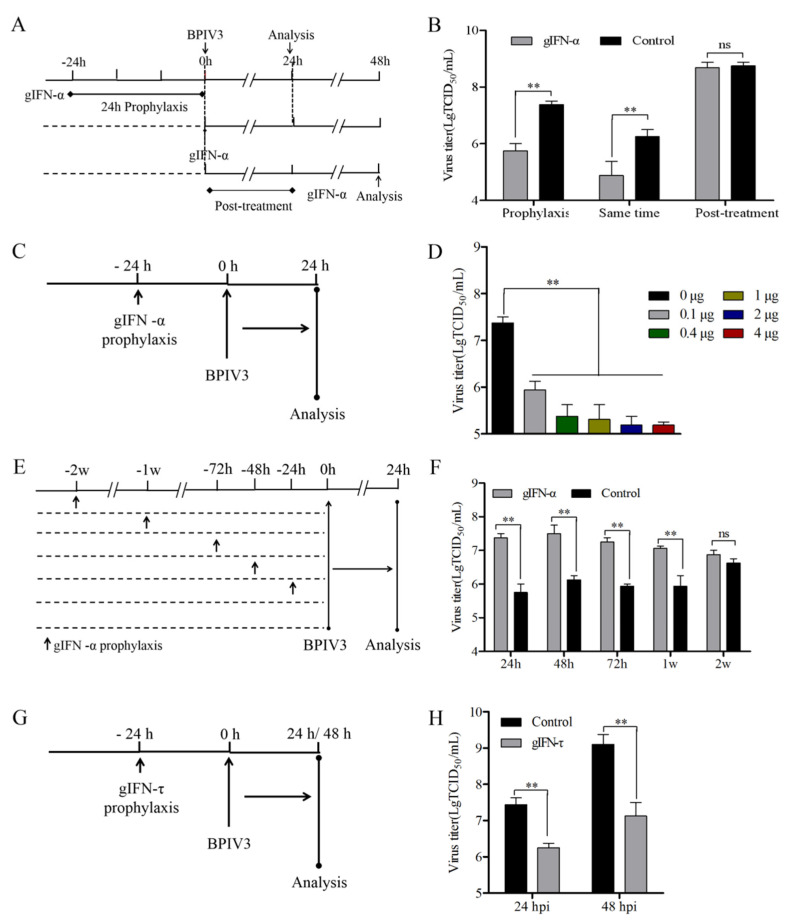
Exogenous goat IFN-α and IFN-τ inhibit BPIV3 replication in MDBK cells. (**A**,**C**,**E**,**G**) Experimental schematic. (**A**) MDBK cells were treated with goat IFN-α (1 μg/mL) 24 h before infection or together with infection, and then the samples were harvested at 24 hpi. MDBK cells were treated with goat IFN-α (1 μg/mL) 24 h after infection, and the samples were harvested 24 h later. The viral titer in untreated cells was used as the negative control. (**C**) MDBK cells were treated with different concentrations of goat IFN-α (0, 0.1, 0.4, 1, 2, or 4 μg/mL) 24 h before infection, and the samples were harvested at 24 hpi. (**E**) MDBK cells were pretreated with goat IFN-α (1 μg/mL), cells were subsequently cultured for varying lengths of time (24 h, 48 h, 72 h, 1 w, or 2 w) prior to infection, and harvested at 24 hpi. (**G**) MDBK cells were pretreated with goat IFN-τ (0 or 1 μg/mL) 24 h before infection, and the samples were harvested at 24 hpi and 48 hpi. (**B**,**D**,**F**,**H**) TCID_50_ results. the virus titers in the culture supernatant between different groups were detected, as shown in the corresponding schematic. The untreated cells were used as the negative control. MDBK cells were infected with BPIV3 at an MOI of 1. The results were reported based on duplicate independent experiments and compared with the negative control in each group. **, *p* < 0.01.

As the antiviral activity of IFNs might differ when challenged with different viruses, we tested the antiviral activity of goat IFN-α and IFN-τ in response to BPIV3, another member of the *Paramyxoviridae* family, cultured in MDBK cells. First, we added 1 μg/mL of exogenous goat IFN-α together with the BPIV3 infection and incubated the mixture for a further 24 h (Figure 6A). This treatment reduced the viral titer by about 24-fold, and the preincubation with goat IFN-α (1 μg/mL) for 24 h reduced the viral titer by 42-fold at 24 hpi, whereas treatment after infection was not effective in modulating BPIV3 infection at 24 hpi (Figure 6B). Furthermore, different concentrations of goat IFN-α were preincubated on MDBK cells for 24 h (Figure 6C), which reduced the viral titer by 27-fold with 0.1 μg/mL goat IFN-α. There was no difference between the groups with different concentrations of goat IFN-α (Figure 6D). These results indicate that BPIV3 infection was also sensitive to goat IFN-α. In addition, the antiviral effect of goat IFN-α in response to BPIV3 infection was maintained for 1 week, with a 13-fold downregulation of viral titer, although this effect was lost 2 weeks after goat IFN-α incubation (Figure 6E,F). Lastly, to further validate our findings, we confirmed the efficacy of goat IFN-τ in modulating BPIV3 infection in MDBK cells. Similarly to CPIV3 infection, administration of goat IFN-τ 24 h prior to infection reduced the virus titer by about 15-fold and 94-fold at 24 hpi and 48 hpi, respectively (Figure 6G,H). 

## 4. Discussion

Type I IFNs were found to be important cytokines in mediating the process of viral infection and other pathogens, and derived diverse subtypes to play antiviral activities with virus specificity [4,11]. In this study, we determined the antiviral activities of goat IFN-α and IFN-τ against CPIV3 and BPIV3 infection in MDBK cells, and provided experimental evidence that goat IFN-α and IFN-τ are potent to suppress viral replication as a prophylactic but not a treatment for viral infection. So far, antiviral activities were only tested against CPIV3 and BPIV3 in MDBK cells. Investigations using other viruses or other models (in vitro or in vivo) need to be performed to consolidate the findings on a broader scale. 

Type I IFNs have essential roles in protecting host cells from virus spread, and the cellular factors that mediate this defence are the products of downstream ISGs. Here, goat IFN-α induced the activation of 550 genes. The ISGs of *IFI6*, *ISG15*, *OAS1Y*, *OAS1Z*, *MX1*, *MX2*, and *RSAD2*, which had been reported to inhibit CPIV3 replication [26,32], were all significantly upregulated in goat IFN-α-incubated MDBK cells. However, excessive activation of the IFN-α pathway might lead to a severe inflammatory response, which enhances the development of the disease [33]. Based on the transcriptome data, goat IFN-α did not induce the production of inflammatory mediators, including IL-1β, TNF-a, GM-CSF, and RANTES—just *IL6* (2.3-fold), *IL11* (2.8-fold), *LIF* (2.2-fold), and *INHBE* (8.5-fold) were slightly elevated, which implies that goat IFN-α induced a negligible inflammatory response. In particular, goat IFN-α, associated with high antiviral effects and low immunomodulation responses, could be used as a drug candidate to help enhance antiviral effects in the host. 

In the present study, we demonstrated that treatment with goat IFN-α after infection was ineffective, whereas goat IFN-α did effectively modulate CPIV3 and BPIV3 infection when administered before infection. CPIV3 nonstructural protein C was detected at 48 hpi in CPIV3-infected cells and antagonized the type I IFN antiviral response [18]. When goat IFN-α was added to an already-established infection, it may have been too late to effectively modulate the course of infection; hence, treatment with IFN-α after infection showed no antiviral activity. The delay of 48 h for CPIV3 to encode the detectable level of nonstructural protein C after CPIV3 infection likely provides a window of opportunity for the activation of the JAK/STAT pathway, suggesting that prophylactic goat IFN-α therapy may close this window of opportunity for the virus. We demonstrated that stimulation of MDBK cells with goat IFN-α before or together with infection effectively modulates CPIV3 infection, and the modulation of infection lasted up to 1 week after goat IFN-α incubation. Thus, the timing of goat IFN-α administration seems to be crucial in modulating respiratory viral infection.

IFN-τ is a type I interferon produced only by ruminants and has been reported to show efficacy in reducing replication of different viruses [34,35,36]. In this study, *IFNT1,* the IFN-τ-encoding gene, was induced by the incubation of goat IFN-α in MDBK cells, and goat IFN-τ was shown to inhibit CPIV3 and BPIV3 replication in vitro, which indicates that *IFNT1* might be a novel direct antiviral ISG in MDBK cells. IFN-τ, like IFN-a, has been reported to activate the phosphorylation of STAT1 and induce the expression of numerous ISGs [34,37], which might also be antagonized by CPIV3 infection. Hence, it proved ineffective on an already-established infection. Meanwhile, pretreated with goat IFN-a or IFN-τ (1 μg/mL) 24 h before CPIV3 infection, both reduced the viral titer by about 115-fold, which showed that they possess similar anti-CPIV3 activity. As to BPIV3 infection, goat IFN-a (27-fold) showed slightly stronger antiviral activity than that of goat IFN-τ (15-fold). This may make goat IFN-t an attractive alternative to IFN-a as a prophylactic during viral infection in the ruminant respiratory tract. 

## 5. Conclusions

We modeled the dynamics of goat IFN-α against CPIV3 and BPIV3 infection in MDBK cells in an attempt to provide insights for future clinical evaluation. In addition, goat IFN-τ is an attractive alternative to IFN-a as a prophylactic during CPIV3 and BPIV3 infection. According to the results of our study, goat IFN-α-based antiviral approaches against CPIV3 and BPIV3 need to be further investigated in vivo.

## Data Availability

Not applicable.

## Supplementary Materials

The following supporting information can be downloaded at: https://www.mdpi.com/article/10.3390/v14051025/s1, Figure S1: Goat IFN-α-induced genes in MDBK cells assessed by qPCR; Table S1: qPCR primers used in this study [38]. Table S2: ISG screen data sets. The table shows complete data sets from ISG screens for goat IFN-α incubation in MDBK cells.
