# Supplementary material for: Temporal Dynamics of the Ruminant Type I IFN-Induced Antiviral State against Homologous Parainfluenza Virus 3 Challenge In Vitro
Source: Viruses. 2022 May 11;14(5):1025. doi: 10.3390/v14051025 (PMC9146716; doi:10.3390/v14051025)
Supplement: Supplementary file 1 [file viruses-14-01025-s001.zip › viruses-1696933-supplementary.pdf]

Supplementary Materials

Figure S1 Goat IFN-α-induced genes in MDBK cells assessed by qPCR.

Table S1 qPCR primers used in this study

Table S2 ISG screen data sets. The table shows complete data sets from ISG screens for goat IFN-α incubation in MDBK cells.

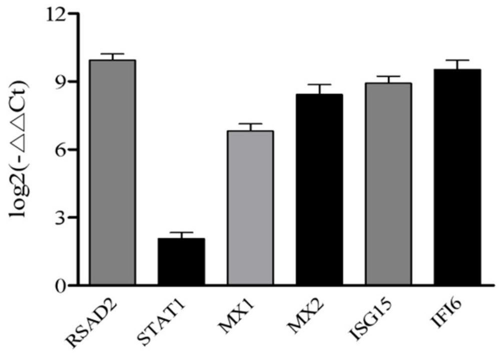

Figure S1. Goat IFN-α-induced genes in MDBK cells assessed by qPCR. MDBK cells were incubated with 1 μg/mL of goat IFN-α for 24 hours, and the untreated cells were used as the negative control. Total cell RNA was extracted, and the mRNA levels were assessed by qPCR. Two independent experiments were performed, and relative quantities of mRNA accumulation were evaluated based on the $2^{-\Delta\Delta Ct}$ method.

Table S1: qPCR primers used in this study.

Gene name	Forward primer（5′-3′）	Reverse primer（5′-3′）	Reference
**RT-qPCR**			
IFNA1	GTGAGGAAATACTTCCACAGACTCACT	TGAGGAAGAGAAGGCTCTCATGA	[38]
IFNB1	CAGATGCCTGAGGAGATGAAG	CTGGTGAGAATGCCGAAGAT	This study
RSAD2	TTCAACGTGGACGAGGATATG	CCAGAGTTCTCACCCTCAATTAT	[18]
STAT1	TTTCCGTTCTGGCTTTGGA	GCTCTCGCTCCTTGCTAATAA	This study
MX1	GCCAACTAGTCAGCACTACATTGTC	GCTCTTGGACTCCATATCTTCAC	[32]
MX2	CTTCAGAGACGCCTCAGTCG	TGAAGCAGCCAGCAATAGTG	[32]
ISG15	GGTATCCGAGCTGAAGCAGTT	ACCTCCCTGCTGTCAAGGT	[32]
IFI6	GTGACAAAGCCTTGAGCTGC	CGCAGGTGTAGAGTAGCAGG	[32]
β-Actin	ACATCCGCAAGGACCTCTA	GATCTCTTTCTGCATCCTGTCC	
**TaqMan RT-qPCR for detecting CPIV3 RNA copies**			
Primers	GCTTGGCTTCTTTGAAATGG	GCCTGCAGAAGTTCCTTGTC	[22]
Probe	FAM-CAATCGGACTAGCCAAGTATGGTGGGA-TAMRA		

Table S2：ISG screen data sets. The table shows complete data sets from ISG screens for gIFN-α incubation in MDBK cells.

Gene-ID	Symbol	log2(fc)	Gene-ID	Symbol	log2(fc)	Gene-ID	Symbol	log2(fc)	Gene-ID	Symbol	log2(fc)
100139670	IFIT1	8.57	515202	USP18	6.05	509678	IFIT3	5.8	281574	EZR	1.09
112441507	Bst2	7.49	517587	INHBE	3.09	507055	GBP4	6.72	507526	CDH17	1.09
280729	B2M	1.51	527520	HERC6	4.87	777788	DDIT3	2.15	533630	PSPH	1.84
280872	MX1	6.5	527528	IFIT2	4.91	613313	GBP2	6.06	281871	ISG15	9.24
280873	MX2	8.87	535490	IFIH1	3.49	513185	PARP12	2.01	509107	ATF4	1.04
281576	WARS	3.17	539807	ZNFX1	3.13	783680	B2M	1.50	112441484	IFITM3	1.85
282255	IFITM3	2.96	540789	PARP14	2.41	533044	PSAT1	1.71	511531	GBP3	3.94
347700	EIF2AK2	2.95	654488	OAS1Y	9.04	101904063	ITGAX	2.57	532684	LOXL2	1.42
497204	UBA7	4.70	784304	CMPK2	6.37	524576	APOL2	2.63	617420	ISG12(B)	1.99
504760	DDX58	3.96	510814	STAT1	1.81	617807	PSMF1	2.16	510933	Aars	1.35
505134	ADAR	2.32	509740	XAF1	4.53	281408	PLAU	1.28	511858	NT5C3A	1.78
506759	IFI16	5.55	415113	HSPA5	1.42	100298356	Bst2	6.99	327662	ANXA1	1.20
508333	ZBP1	6.30	777594	IFITM3	2.21	508877	PNPT1	2.06	513605	GTPBP2	2.19
509283	RNF213	3.46	781914	CMTR2	2.43	281476	SARS	1.55	508861	SAT1	1.036
509859	TRANK1	2.25	515712	HLA-A	1.67	347699	OAS1X	9.20	100125591	IRF7	2.2
510532	PARP9	2.41	506415	RSAD2	9.67	282355	SLC1A5	1.97	338039	CASP13	1.53
512486	GBP3	4.23	510377	SP140	2.42	100297676	Clec2h	2.03	782274	TNFRSF10A	1.17
512913	IFI6	4.14	505103	PHGDH	2.11	505265	TRIM5	1.24	614589	ZC3HAV1	1.27
514205	SAMD9	6.31	408010	GARS	1.63	521795	SLFN11	1.25	525795	AGRN	1.16
514373	HERC5	5.22	514896	TRIM56	1.43	533038	TM4SF1	1.14	513572	ERAP2	1.61
515051	DTX3L	2.99	510991	PARP10	2.51	524959	TAP1	2.02	514209	ASNS	1.77
281528	TGM2	1.94	532442	RTP4	3.51	539159	CTH	2.55	509855	IRF9	2.19
515091	IFIT5	2.07	614555	Epsti1	3.8	508378	DHX58	6.89	514386	BIRC3	1.49

Gene-ID	Symbol	log2(fc)	Gene-ID	Symbol	log2(fc)	Gene-ID	Symbol	log2(fc)	Gene-ID	Symbol	log2(fc)
533049	HLA-A	1.21	539820	TRIM34	1.73	509863	SESN2	2.08	533992	DRAM1	1.09
280701	PPA1	1.35	512531	MARS	1.03	282077	SPARC	1.49	512672	Patr-A	1.43
517417	XRN2	1.20	506029	STRADB	1.23	512184	CDADC1	1.22	282013	PSMB8	1.103
407173	HLA-A	2.00	104973909	ERVPABLB-1	2.12	616948	TRIM5	1.51	507781	NEDD4	1.04
513281	CPM	1.63	516864	ALDH1L2	2.26	506604	ISG20	2.93	518469	GPCPD1	1.51
515204	SP110	2.16	513479	RNF114	1.23	616168	ICAM5	1.01	282711	EPAS1	2.15
538519	MKNK2	1.49	614776	POLR2K	1.25	524683	SAMHD1	1.17	507138	IFI27	9.93
100125267	LIPA	1.31	618042	VGF	2.42	513220	MKI67	1.24	539781	Chmp5	1.01
514739	MAP1B	1.16	507215	TNFSF10	6.04	790880	ATXN3	1.08	100139208	SP140L	2.28
511023	STAT2	1.19	282295	HYOU1	1.10	524531	USP25	1.25	510593	PSMB9	2.86
282856	PCK2	1.79	505940	ARHGEF2	1.16	539141	PSMA2	1.06	617530	CEBPG	1.10
509620	CMTR1	1.42	280981	PLIN2	1.56	513231	GTF2B	2.19	614242	RAB8B	1.22
517535	HSPA9	1.02	540584	FAM126A	1.30	281581	YARS	1.10	100137953	ATP8B4	6.34
100138545	PML	1.23	508269	TMEM106A	3.37	507197	SHMT2	1.04	530999	Clstn2	1.18
508347	IFI44L	9.27	524078	Slc7a11	2.26	510813	LGALS9	7.50	100337295	IL11	1.47
616886	IGFBPL1	1.40	508348	IFI44	9.30	514787	STK38L	1.41	504507	TNFSF13B	1.01
510417	HLA-A	1.76	614655	DESI1	1.08	515475	TMEM140	2.63	513308	ULBP1	1.41
506045	ATAD1	1.20	617047	TCIM	2.43	615833	IFITM1	1.69	508638	NOC2L	1.07
280686	F3	1.47	101902412	ANGPTL8	2.87	532944	LHFPL6	1.50	614093	PXK	1.07
531137	LGALS3BP	1.02	539867	SYT4	1.57	533050	HLA-A	1.13	522469	BATF2	4.32
359715	TRIM21	1.48	507107	SLC3A2	1.26	515715	Cars	1.13	787569	LARP6	1.09
538465	TRIB3	2.32	510807	NFE2L3	1.05	541283	IFRD1	1.28	781648	LAP3	1.14
326577	SLC1A4	1.72	510748	PLEKHA4	2.27	539087	RYDEN	1.33	100336669	GBP7	5.09
511328	B4GALT4	1.74	614497	AKAP2	1.06	512633	MFSD2A	1.4	504336	DAXX	1.17
520472	NAMPT	1.33	517539	MTHFD2	1.82	538691	HS3ST1	2.83	529235	DDIT4	1.15

Gene-ID	Symbol	log2(fc)	Gene-ID	Symbol	log2(fc)	Gene-ID	Symbol	log2(fc)	Gene-ID	Symbol	log2(fc)
518368	PARM1	9.17	617625	RBM43	1.25	112445390	TNFRSF10A	1.11	617105	EEF1E1	1.14
787099	ZCCHC2	1.90	504467	SASS6	1.19	282005	PROCR	1.94	767904	MGC126945	2.33
618409	IRF4	6.73	522632	TRIM14	1.61	787696	TOP2A	1.04	539960	STX11	1.21
527214	PLA2G12A	1.31	353510	IFITM1	4.47	512905	APOL2	2.46	533681	TMEM156	2.21
281983	PLAUR	1.30	507917	HLA-A	1.1	786701	MUC4	2.96	615277	them4	1.08
533089	CENPF	1.43	112444847	IFITM1	4.69	514346	SDS	1.95	512637	ADGRF1	1.25
535202	SLCO5A1	1.64	281375	SERPINE1	1.03	782472	CGAS	3.14	281970	PDE1B	1.49
510697	IFI35	1.37	540142	FOXS1	6.48	782690	Tcf7	1.04	516949	GBP5	3.75
526873	CAMK1D	1.15	526945	ARHGAP19	1.93	534697	KLHL7	1.37	526331	GALNT16	1.28
785790	LEKR1	2.66	282299	PAFAH2	1.51	538529	SLC25A28	1.54	282127	ZFP36	1.02
531022	TRIM38	1.21	788334	APOL2	2.43	508271	DSN1	1.14	529660	OAS2	9.62
513497	CDKN1A	1.17	539606	PYCR1	1.41	527854	KIF20A	1.50	539274	AKNA	1.24
537810	AKAP6	2.08	504400	RBCK1	1.18	506932	CHST1	1.96	512133	DCSTAMP	1.25
100140338	SP100	6.39	614212	LMO4	1.1	533338	BCL2L12	1.18	282369	SLC7A5	1.41
514701	Fam3b	6.41	101904723	CCDC194	5.18	509161	DLGAP5	1.49	540206	MASTL	2.47
787488	AFF3	1.60	281193	GJA1	2.56	534910	CD200	1.44	407121	NEB	1.13
521254	BPNT1	1.26	507439	NMUR2	3.13	541166	Etv5	1.84	280840	LIF	1.11
505991	CHAC1	2.79	504225	AKIP1	1.04	518030	TAF4B	1.85	104968609	Slc9c1	4.25
533862	PAPD4	1.14	515697	APOL2	3.45	528939	FAM160A1	1.11	100850276	ULBP3	1.96
540135	Phlda1	1.40	509471	UBE2L6	2.42	505316	MISP	1.29	785576	FGF21	3.7
519922	OAS1Z	9.48	282099	TREX1	1.64	508763	TFPI	1.23	516599	TRIM5	3.44
788454	MAGEB4	2.09	531254	COL24A1	1.01	540874	DEPDC1B	1.21	282855	PCK1	3.92
541284	MBOAT1	1.23	282589	HSD11B1	1.24	782542	ONECUT2	1.09	613705	Rbmx2	1.04
511037	TMPRSS2	2.36	510276	RASSF1	1.29	512660	SEMA3C	1.06	100336228	FAM71E2	1.67
507550	Stra6l	1.77	533724	ZUFSP	1.08	509780	PRICKLE3	1.43	784362	SCEL	1.48

Gene-ID	Symbol	log2(fc)	Gene-ID	Symbol	log2(fc)	Gene-ID	Symbol	log2(fc)	Gene-ID	Symbol	log2(fc)
512869	RNF213	2.91	516921	EML6	1.14	538846	JAM2	1.42	786352	APOL2	8.68
510774	ABHD1	3.86	507960	ARHGAP9	1.23	510699	RND2	2.08	280847	LUM	5.04
613633	SPRED3	1.18	534781	PBK	1.52	614322	RUNDC3A	1.47	282363	SLC6A2	2.55
614675	PDLIM1	1.33	616503	Rras	1.07	282043	RPE65	1.76	533161	KIF2C	1.2
767910	PLAC8	9.43	281386	SERPINF1	3.72	281667	CCNA2	1.19	507731	CDC25C	1.09
615030	IL15RA	1.16	100295656	Gm525	1.64	508043	PTTG1	1.39	521399	FAM83A	1.10
504932	TMEM190	1.32	112443864	MICA	1.65	507498	CKAP2L	1.41	615226	GRIK2	1.06
517232	RECK	1.63	516769	A2ML1	1.25	788414	ESRP1	1.13	524925	TTK	1.10
281346	NCF2	1.61	327679	CCNB1	1.18	518597	MAB21L3	1.12	540846	ETV1	2.65
783855	TIFA	2.39	539998	FBXO33	1.06	506740	ESPL1	1.06	531747	ADCY10	1.08
538771	KIF5C	5.79	515376	CDC20	1.45	510523	TMEM154	1.03	507988	CYP3A24	1.93
537688	Prps2	1.04	511654	CHAF1B	1.34	101906312	HAVCR2	1.34	281702	CNGB1	5.13
613606	AKAP7	1.68	414922	CLDN1	1.52	509786	BCL2L15	3.92	529166	CBLN3	2.28
617456	HJURP	1.36	539780	NAIP	1.03	533834	CD274	3.87	504585	SPAG5	1.37
537027	BUB1B	1.03	613954	RAB37	1.68	538521	TGM7	4.35	505078	APOL3	4.25
528870	NAV3	1.06	616942	HLA-A	1.17	768028	BEX2	1.06	539464	PCDH18	1.16
282521	SERPINE2	1.04	280712	ADA	1.1	281797	GNGT2	9.26	280826	IL6	1.19
505886	IQGAP3	1.08	282161	CACNB4	1.18	617024	E2F2	1.21	281357	NPR2	2.089
539153	SLC16A14	1.83	281376	SERPINB2	9.94	618737	BST2	8.24	504282	PLK3	1.25
616194	HSPB11	1.25	504437	AURKA	1.42	527651	WDR25	1.50	281668	CCNB2	1.04
526279	CASP7	1.33	505791	TROAP	1.41	786852	CIT	1.14	104968404	Nat6	2
100296226	PMAIP1	3.77	527397	DEPDC1	1.06	613895	TEX30	1.01	530913	APH1B	1.2
533985	RHBDD1	1.43	100337356	NAALADL2	1.22	768246	Fbxo16	1.25	614434	CDCA3	1.17
506962	UBE2C	1.41	523367	SHCBP1	1.51	618412	Dusp15	1.16	408005	HEY1	1.24
534849	ASPM	1.31	407998	F11	1.81	616028	NUSAP1	1.05	529868	TICRR	1.3

Gene-ID	Symbol	log2(fc)	Gene-ID	Symbol	log2(fc)	Gene-ID	Symbol	log2(fc)	Gene-ID	Symbol	log2(fc)
785408	ZFP28	1.18	786516	CRB2	1.35	506390	EXO1	1.09	539741	RNASE13	2.34
100298064	HAVCR1	1.74	616063	MYADM	3.30	521656	CYP2J2	2.02	790225	MLKL	1.94
101903126	GVINP1	1.75	530407	Neto1	1.95	530961	BARD1	1.14	531014	SPTBN5	1.70
781710	GBP2	9.31	514203	GHSR	4.91	531482	MORN3	1.1	516073	THEM6	1.11
281004	APOE	2.22	525750	TTC32	1.30	518795	SOCS1	1.3	615857	PRR11	1.28
518752	ARG2	1.01	525027	PINX1	1.13	528149	CHSY3	1.53	538515	FAM107A	1.05
618289	ZNF267	1.25	539573	MEIS2	1.87	505080	TGM3	3.35	615288	SELENOV	1.47
515741	CAPN14	2.42	615922	Ccdc136	1.38	526769	PTPN11	1.77	101904203	C4orf54	3.91
525695	LVRN	2.78	100336572	ARSH	1.01	517588	GLI1	1.04	281535	TLR3	1.06
616148	LBH	3.14	511170	KIF18B	1.51	540455	PIMREG	1.55	104968627	FSIP2	2.86
781494	MYADM	2.14	108771180	APOBEC3H	1.29	510973	NUMBL	1.10	526515	ARMC4	1.82
107132081	MAGEB2	1.28	327703	CACNB1	1.07	509575	JAML	3.32	506708	RADIL	1.19
613724	DYNC1I1	1.17	281914	MMP13	3	508394	CASQ1	1.20	100336077	PRSS48	1.66
617315	FAM217A	5.75	514971	Cyp2s1	1.98	508153	MYADM	2.61	540145	FBXO43	1.12
514463	TEKT2	1.59	450212	RXFP4	1.70	281904	LOXL4	1.87	615282	CDKN3	1.55
787891	CCDC187	1.08	781188	MRAP2	8.80	506294	KIF22	1.07	523830	GAL3ST2	6.41
613605	KCNQ5	2.83	100301478	CCNB3	1.43	539571	ESM1	1.29	107132327	CYP2J2	4.86
337925	SLC8A1	2.47	523998	NEK2	1.21	517231	RUBCNL	1.58	539935	GJB3	1.20
518234	STAC3	1.25	788092	DNAH17	3.7	616082	DUPD1	2.12	509065	RGSL1	1.44
101907121	RTL4	1.21	512408	RAD21L1	1.36	615438	BTBD19	1.12	515679	NUGGC	3.77
532898	TNS4	1.81	507688	GLDC	1.1	617445	KAZALD1	1.42	534432	CCDC39	2.7
100336873	LAMA3	1.86	781721	C16H1orf21	1.4	614840	GAS2	1.55	618054	PROM1	2.91
100336423	Oscar	3.97	616965	FAM72A	1.96	407133	TACR1	2.58	786258	RAN	2.58
286849	CD40	1.51	101906350	CCDC168	1.05	514509	AGMAT	1.71	522726	NT5DC4	1.74
540119	MCHR1	2.52	613414	TRH	1.02	526674	MYH15	2.25	518896	ADD2	1.02

Gene-ID	Symbol	log2(fc)	Gene-ID	Symbol	log2(fc)	Gene-ID	Symbol	log2(fc)	Gene-ID	Symbol	log2(fc)
616691	CATSPERE	4.81	281174	FUT1	3.73	MSTRG.10329	--	2.96	MSTRG.2885	--	1.03
541135	Kif15	1.05	101904283	LY6L	1.09	MSTRG.5305	--	1.63	MSTRG.6476	--	1.97
516291	Rec8	3.86	112445660	IFNT1	1.33	112446427	--	1.98	MSTRG.891	--	2.2
101903854	Spef1	1.21	520341	TNFRSF9	2.38	MSTRG.13667	--	1.65	MSTRG.8192	--	1.3
520712	C22orf23	1	503554	COX6B2	2.33	107132617	--	2.77	MSTRG.7959	--	1.80
533129	ETV4	2.06	526230	Cp	1.17	MSTRG.5856	--	2.66	MSTRG.8704	--	1.34
507646	Tm4sf4	2.47	281644	BFSP1	1.27	MSTRG.536	--	11.35	112448863	--	2.73
789769	SH2D5	1.38	506132	CH25H	1.58	MSTRG.14369	--	1.44	112449284	--	1.53
522763	MYADM	3.12	616348	ARL14EPL	1.97	MSTRG.3683	--	1.19	112442374	--	1.46
280969	ABAT	1.76	526745	fer-1-like protein 4	1.71	MSTRG.7203	--	1.27	112447334	--	3.28
506315	Dnm3	1.18	783920	GTPase 1-like	3.94	MSTRG.9440	--	1.47	MSTRG.8425	--	1.37
511034	TMEM108	1.52	MSTRG.3546	pol	1.63	MSTRG.4762	--	1.18	MSTRG.3025	Pol	1.73
783508	ULBP2	1.56	MSTRG.12357	Patr-A	1.95	MSTRG.13619	--	5	MSTRG.7765	pol	1.55
504548	UBD	1.90	MSTRG.7126	gag-pro-pol	1.06	101903477	--	1.28			
281204	GNG3	1.39	MSTRG.13657	NCR3LG1	2.74	MSTRG.7465	--	1.50			

Annotation: log2(fc) means the log2 (fold change).
